# Preventing Children From Developing Dyslexia: A Premature Writing Hypothesis

**DOI:** 10.1177/00315125221075001

**Published:** 2022-03-02

**Authors:** David S. Mather

**Affiliations:** 1 Department of Curriculum and Instruction, 8205 University of Victoria, Victoria, BC, Canada

**Keywords:** callosal agenesis, corpus callosum, dyslexia, eye dominance, handwriting, Hebrew, maturation, right hemisphere

## Abstract

It has been argued that dyslexia may develop in strongly left eye dominant children through learning to write using ipsilateral, right hemisphere motor pathways. New light on this theory has been cast by recent findings of atypical enhanced corpus callosum white matter in children with dyslexia, reflecting right to left hemisphere communication that is resistant to intensive remedial reading intervention. Enhanced corpus callosum white matter is consistent with uninhibited right to left hemisphere ipsilateral mirror-motor innervation, manifested as frequent mirror-letter writing errors in children with dyslexia. Delaying writing instruction until 7–8 years of age may prevent these errors and as well as the development of dyslexia. During the 7–8-year age period, visual-proprioceptive integration enables a child to mentally map whole word visual images onto kinesthetic/proprioceptive letter engrams (memory representations). Hypothetically, this process is facilitated by anterior commissure activity involving interhemispheric transfer of ipsilateral mirror-to-non mirror-motor movement. This postulate, involving delayed writing instruction pending further maturation, also receives indirect support from the remarkable proficiency leap among second graders reading Hebrew as Hebrew involves a leftward orthography in which ipsilateral right to left hemisphere innervation is uninhibited. Additionally, and more directly, normal reading comprehension for learning English among children with agenesis of the corpus callosum suggests that letter-sound decoding is not the sole route to proficient reading comprehension. In this paper, I make recommendations for obtaining empirical evidence of premature writing as a cause of dyslexia.

## Introduction

The central argument of this review is that left eye/right hemisphere (RH) dominant beginning writers of English, in learning to write letters, must cope with ipsilateral RH-to-left hemisphere (LH) corpus callosal innervation that is kinesthetically reversed in order and orientation to the rightward direction of print. In right eye/LH-dominants, ipsilateral mirror-innervation is inhibited through letter-sound decoding, but in left eye/RH-dominants it is not and consequently strong RH dominance induces sequential letter (e.g., was/saw) and spatial letter (e.g., b/d) mirror-writing that confuses and interferes with normal reading development. Research findings of enhanced RH-to-LH corpus callosal communication in dyslexic readers are consistent with such ipsilateral mirror-motor interference. However, there is considerable evidence that the visual system may be able to remap mirror-reversed kinesthetic innervation onto conventionally ordered print if letter writing instruction is postponed until 7–8 years of age. Studies that have found normal reading comprehension among individuals with congenital absence of the corpus callosum (full or partially absent corpus callosum), provide support for access to whole word LH processing via anterior commissure, mirror-to-non mirror interhemispheric communication. This likelihood is further indicated by the Hebrew reading leap at age 7–8 from spelling-to-sound translation to whole word processing, since written Hebrew is a left-to-right orthography in which RH-to-LH ipsilateral mirror-innervation is uninhibited.

In English writing, uninhibited ipsilateral mirror-innervation is first manifested by the spontaneous mirror-writing of children below the age of 8 years. For example, Cornell (1985) found that 82% of 5-year-old children, irrespective of their sex or handedness, printed their name conventionally (canonically) to the right and in mirror-form to the left of a vertical line bisecting their writing paper. They were unable to discern any difference between their canonical and mirror-image productions—a difficulty shared by unschooled (illiterate) adults (Kolinsky et al., 2011; Pegado et al., 2014). Using Cornell’s protocol, kinematic research with four- and 5-year-old French children found that both mirror and conventional characters were written with equivalent ease (Portex et al., 2018).

The mirror-writing of Cornell’s (1985) participants is consistent with ipsilateral movement control: Muller et al. (1997) found that ipsilateral motor pathways in typically developing children are not fully inhibited before age 10 and there is evidence that these pathways are recruited by mirror-writing (Elder, 1897; Ellis, 1988; Vaid & Carr, 1999). Peripheral visual processing may also be involved in mirror writing, as 5-year-old (vs. 8-year-old) children do not automatically process foveal stimuli before peripheral stimuli (Holmes et al., 1977). For example, Aaron and Malatesha (1974) found that visually discriminating mirror-image letters and shapes at 3.8° to either side of eye fixation was better at short (80 msec) exposures than at long (3.5 sec) exposures in six- and 7-year-old, but not in 8-year-old, boys. This discrimination improvement is coincident with the disappearance of spontaneous mirror-writing by 8 years of age (Brennan, 2012; Chapman & Wedell, 1972; Cornell, 1985).

**Figure 1.**
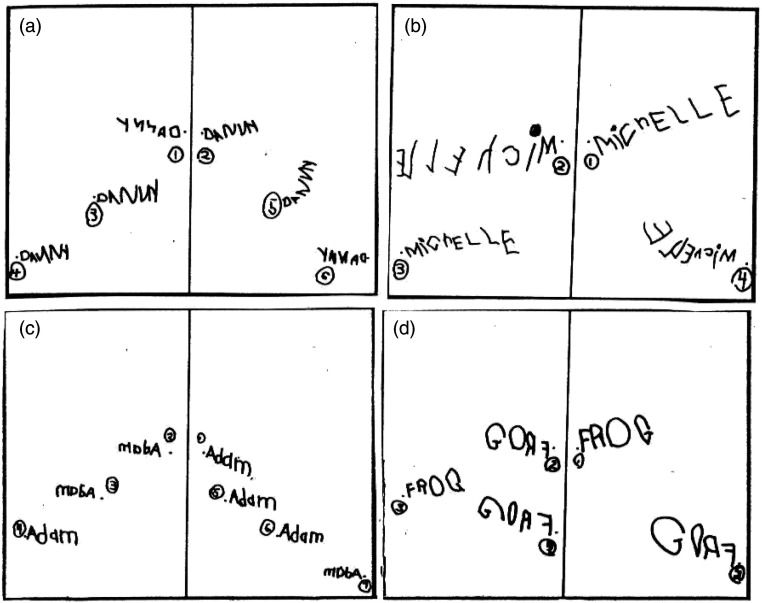
Left Hemispace Mirror-Writing and Right Hemispace Canonical-Writing by Four Primary School Students. Reprinted from https://blog.apastyle.org/apastyle/2016/01/navigating-copyright-part-2.html

Mather (2011) hypothesized that the spontaneous mirror-writing reported by Cornell (1985) reflected a proximal stage of upper limb motor development during which either side of the brain can participate in writing—the left hemisphere (LH) in canonical form and the right hemisphere (RH) in mirror form. Mather (2011) further suggested that RH-dominant beginning writers would naturally manifest RH, rather than LH, mediated proximal motor control, and this would result in the inverse hemispatial writing pattern seen in Figure 1. That is, letter engrams (mirror-memory representations) in left hemispace would not correspond to the visual letter stimuli, and thus would require canonical conversion. Mather (2011) found that many children (39%) produced canonical letter writing in left hemispace. This is close to the percentage of individuals with left eye dominance in the general population (Coren, 1974; Ehrenstein et al., 2005; Fagard et al., 2008; Porac & Coren, 1976) and close to the percentage of elementary school children who sequence animal pictures in a horizontal array of rectangles from right to left (Johnson, 1990). It is also close to the percentage of five- and 6-year-old children that Laszlo and Bairstow (1984) found to be incompetent in kinesthetic acuity, perceptual ability, and memory.

Subsequently, Mather, et al. (2015) argued that most left eye/RH-dominant children suppress mirror-letter confusion, accounting for the atypical global word processing found in some proficient readers (cf. Pugh et al., 1997; Welcome, et al., 2009; Welcome & Joanisse, 2012). For example, Frith (1980) found that early adolescents, who read well but spelled poorly, neglected interior letters in words in both reading and spelling, as though “reading by eye and spelling by ear” (p. 512). Compatibly, a recent whole-brain fMRI study found that, despite degraded orthographic representations, children with isolated spelling deficits showed no reliable reading-related fMRI neural network differences from typically developing children. In contrast, children with dyslexia showed lower neural network brain activity due to their difficulties with both sub-lexical and lexical processing (Banfi et al., 2020). However, for a minority of left eye/RH-dominant beginning writers, possibly due to unusually strong left eye dominance (cf. Chaumillon et al., 2017), dysfunctional RH-mediated letter encoding may account for the longstanding association between left eye dominance and reading disability (Dearborn, 1931; 1932; Denckla, 1974; Monroe, 1932).

## Dyslexic and Typical Reading Development in Children Learning English

### Letter Processing in Dyslexia

There are two major subtypes of dyslexia: In the phonologically deficient subtype, letter processing is encoded holistically by shape whereas, in the phonologically proficient subtype, it is encoded analytically by sound (Lachmann & Van Leeuwen, 2008). In the latter group, there is an early tendency to reverse palindromic words (e.g., was/saw) (Gjessing & Karlsen, 1989; Johnson & Myklebust, 1967). For example, Schevill (1978) found that right-handed reading-disabled primary school children with deficient spatial memory, when asked to identify words traced out-of-sight on their left palm, pointed to the visual representation “on” when matching the traced letter sequence “n-o.” These errors differ from the spatial reversals (b/d) that are significantly more common in phonologically deficient readers, for whom letters appear to be encoded as pictures rather than phonetic units (Aaron, 1978; see also Brem et al., 2013). The two error types have been designated, respectively, as “kinetic” and “static” reversals (Orton, 1931, p. 173). Sidman and Kirk (1974) found that, in seven- to 14-year-old children with reading problems and static reversal errors, a delayed matching task (i.e., matching a sample “b” to a lowercase or uppercase comparison stimuli “b” or “d” after the sample has been removed from sight) increased the probability of b/d letter reversals, indicating an over-reliance on visual memory (Terepocki et al., 2002).

Almost 100°years ago, Orton (1925) attributed letter-reversal errors among school children experiencing unusual reading difficulty to “. . . confusion, because of reversals, in the memory images of symbols resulting in a failure of association between the visually presented stimulus and its concept.” (p. 610). Subsequently he added “… the confusions so arising do not rest on disturbances in the visual process but rather on an ambivalence or variability in the engrams with which the visual experience is to be compared. . .” (Orton, 1931, p. 166) and that “… there has been a failure to establish the normal facile association between the visual and the kinesthetic engrams, resulting in a measure of conflict” (Orton & Gillingham, 1933, p. 24). Analogous visual-kinesthetic conflict was observed in a series of studies, summarized in Young et al., (2015), showing atypically reversed rotation actions in children with written language difficulties. These actions included unscrewing bottles in an unusual way and, when drawing clockwise circles, sensing that the pencil was moving counterclockwise. Young et al. called this Reversed Positioning Sensation (RPS) and postulated that RPS impaired written language learning because the movement sensation during writing was mirror-reversed to the visual input of the letter being formed. This is comparable to Mather’s (2011) description of children’s tendencies to confuse conventional letter forms with their mirror-memory representations (engrams). Further support for this hypothesis stems from evidence that children with written language difficulties have problems drawing continuous garland figure eight loops without strong visual control, tending to reverse the loops in the opposite counterclockwise or clockwise direction to that intended (Brown, 1990, p. 294. Fig. 18.3). However, Brown also found that, after blindfold practice with the hand guided by a teacher and gradually withdrawn, these children were able to kinesthetically produce the loops on their own without the blindfold. Moreover, they no longer produced mirror-reversal letter errors. Similarly, in teaching cursive writing to children with handwriting problems, Benbow (1995) found that letters beginning with a clockwise stroke often induced mirror-reversals and that averting the gaze assisted children to properly write letters with abrupt across-midline directional change. Reversed kinesthetic letter engrams may also account for findings that some poor writers are unable to identify letters with their eyes closed when their hand is guided (holding a stylus or pencil) in tracing the letter shapes (Orton & Gillingham, 1933). Finally, in children with reading disorders, Goulème et al. (2019) found that subjective visual vertical perceptions were significantly better under counterclockwise than clockwise tilt conditions; and Vieira et al. (2013) found that proprioceptive circle centering performance was more accurate in counterclockwise exploration.

## Left hemisphere Inhibition of Right Hemisphere Involvement in Typical Young Readers

Letter-reversal confusion is not a problem for typically developing readers. Evidently, RH ipsilateral mirror-motor innervation is inhibited by LH mediated letter-sound decoding. This is marked by increased corpus callosum isthmus thickness in 6–8-year-old children (Westerhausen et al., 2010), reflecting a decrease in the transfer of information from the RH to the LH. It is also associated with increased phonological decoding efficiency (Dougherty et al., 2007). Contralateral LH inhibition of RH ipsilateral mirror-motor innervation is further indicated by left hand mirror-motor phenomena in right-handed adults following incapacitating right arm damage (Schott, 1980). Among these persons, door knobs or screwdrivers are turned in the opposite direction to that intended, and there is an inclination to reverse letters and write in a leftwards direction with the left hand. These observations led Schott to write, “It would seem therefore reasonable to propose that all movements, including those such as writing which are linguistically related, may be executed as bilateral and homologous movements—with the unwanted side being inhibited or suppressed, as Westphal (1875) first proposed over a century ago.” (p. 772). In this view, kinetic (“was/saw”; “on/no”) and static reversals (b/d) in dyslexia may, respectively, represent LH mediated verbal-sequential and visual-spatial attempts to cope with unwanted RH ipsilateral innervation that is reversed in order and orientation to the direction of print. Correspondingly, children with dyslexia have been found to rigidly apply either an analytical or a holistic strategy to the perception of letters (Schmitt et al., 2019).

Consistent with uninhibited RH ipsilateral innervation in dyslexia, the structure of the posterior corpus callosum has been found to differ in both children and adults with dyslexia (Duara et al., 1991; Rumsey et al., 1996; von Plessen et al., 2002). Correlations between diffusion tensor imaging (DTI) properties within posterior callosal regions and reading-related skills indicate enhanced interhemispheric communication in dyslexic readers compared with typical readers (Dougherty et al., 2007; Frye et al., 2008; Hasan et al., 2012; Odegard et al., 2009). Huber et al. (2018) found this to be remarkably stable in struggling readers (ages 7–12) during an 8-week, intensive phonological decoding reading intervention that produced large changes in tensor imaging diffusion properties throughout a collection of cortical association and projection tracts. Based on this evidence, Huber et al. (2019) postulated that “anatomical differences that are stabilized prior to age seven (the youngest individuals included in our sample) in the posterior callosal tract may ultimately shape reading development. . .” (p. 7). In agreement, a review of brain network dynamics among people with dyslexia concluded that “callosal maldevelopment” is “centrally implicated” in dyslexia (Kershner, 2016, p. 28). Zhao et al. (2016) suggested that the increased interhemispheric neural connection in people with dyslexia results from atypical cerebral dominance, that compromises the two LH white matter tracts that normally participate in orthographic-semantic/phonological and visuospatial processing, In line with this view, and on the basis of bilateral visual spatial and letter/shape dichaptic tests, Witelson (1977) concluded that people with developmental dyslexia may read predominantly with a spatial-holistic, rather than phonetic-sequential strategy. Congruently, Witelson titled her paper: *“Developmental dyslexia: Two right hemispheres and none left.*” Putatively, this spatial-holistic strategy was due to enhanced RH-to-LH callosal communications. DTI posterior callosal tractography may offer a means of identifying this maldevelopment before it becomes irreversible.

### Visual-Kinesthetic/Proprioceptive Integration in Typical Development

In the present view, enhanced RH-to-LH corpus callosum communication in dyslexia results from left eye/RH-dominant children learning to write via an uninhibited ipsilateral RH-to-LH motor pathway in which kinesthetic innervation is mirror-opposed to both the order and orientation of letters in print. Hypothetically, this mirror-conflict may be avoided by postponing the introduction of writing. The age of 8 years is a developmental milestone for the integration of vision with proprioception (a term that is interchangeable with kinesthesia; Stillman, 2002). Children under 8 years of age rely more on proprioception than vision (Hay et al., 1991; Lantero & Ringenbach, 2007; Manyam, 1986; Redon et al., 1994) and there is increasing sensitivity to visual-proprioceptive asynchrony between 5-8-years-of-age (Jaime et al., 2014). For example, 8-year-old, but not 6-year-old children are able to map proprioceptive space onto visual space in a line-drawing visuomotor adaptation task (Contreras-Vidal et al., 2004). As well, in a mirror-drawing task in which 5-to-6-year old and 7-to-8-year-old children practiced tracing a square while looking at the reflection of their hand in a mirror, only the older group was able to re-calibrate their perceptual and sensorimotor systems to learn the required visual-proprioceptive mapping (Julius & Adi-Japha, 2016). These studies suggest that, in children at risk for developing dyslexia, vision may be able to remap mirror-reversed kinesthetic/proprioceptive letter engram innervation onto conventionally ordered print, if letter writing instruction is postponed until 7–8 years of age.

## Reading Development in Individuals with Agenesis of the Corpus Callosum

Additional support for mirror-to-non mirror-letter engram mapping stems from studies that have examined reading development in individuals with agenesis of the corpus callosum (AgCC). AgCC is a rare condition, caused by genetic or environmental factors during prenatal callosal development, in which callosal fibers are completely or partially absent. However, interhemispheric communication via the anterior commissure (AC) is usually not affected (Paul et al., 2007). Comprehensive studies of two normally intelligent, early adolescent children with AgCC found age-appropriate sight word decoding and reading comprehension but phonological difficulties with nonword reading, written word rhyme judgment and spelling accuracy (Temple et al., 1989; Temple & Ilsley, 1994). This suggests that sub-lexical phonological awareness, although considered critical for typical reading development (Tijms et al., 2020), is not the only path to proficient reading comprehension.

Consistent with corpus callosum absence and impaired phonological awareness, in AgCC there is no inhibition of RH ipsilateral innervation and no typical corpus callosum isthmus growth (Genç et al., 2015). However, this does not give rise to ipsilateral mirror-movement phenomena (Ocklenburg et al., 2015), arguably because of AC mirror-to-non mirror, interhemispheric engram transfer. Corballis (2018) argued that “[t]he anterior commissure may be especially involved in mirror-image equivalence” (p. 5) and “may be specialized for memory transfer, making little contribution to perceptual transfer” (p. 6). In line with this view, Thut et al. (1997) demonstrated, in adult participants with AgCC (AC intact), a left hand to right hand faculty for mirror-memory transfer of two-cm-high letter drawings that was inhibited in neurotypical controls. These authors concluded that “[s]ince non-dominant-to-dominant distal transfer was. . . disadvantageous in healthy subjects, the patients’ relative superiority in this condition may reflect missing callosal influences of an inhibitory nature” (Thut et al., 1997, p. 365).

AC mirror-to-non mirror transfer may also provide letter engram access to whole word visual representation in the ventral anterior temporal lobe (ATL) of the LH. The ATL is directly connected to the AC (Catani et al., 2002; Demeter et al., 1990; Schmahmann & Pandya, 2006) and the ATL has receptive fields that cover substantial bilateral portions of the visual field. It plays a key role in whole word (exception/irregular) reading in both ideographic (Japanese Kanji) and alphabetical scripts (Joyal et al., 2017; Ueno et al., 2018), and the ATL is activated by reliance on semantic rather than phonological processing (Hoffman et al., 2015). AC-ATL whole word-letter engram access may therefore account for the previously noted normal reading comprehension in adolescents with AgCC. On the other hand, an absence of ATL access may account for differences between the two major subtypes of dyslexia in which poor phonological decoding is associated with good reading comprehension and good decoding proficiency is associated with poor reading comprehension (Catts et al., 2006). In the latter, poor reading comprehension is manifested by difficulty decoding irregularly spelled (exceptional) whole words that are typically read with semantic support (Nation & Snowling, 1998b; Ricketts et al., 2008). Conversely, good comprehension dyslexia relies heavily on semantic support in reading (Nation & Snowling, 1998a), presumably via peripheral whole word ATL access (see below; Geiger & Lettvin, 1987).

Among individuals with AgCC, foveal whole word access has been demonstrated by briefly presenting six-letter words made up of 3-letter pairings in their composition (e.g., MAN-AGE, ROT-ATE), as this permits symmetrical straddling of the midline between the LH and RH hemifields of vision (Sperry, 1968). In Corballis and Finlay (2000), a 39-year-old woman and her 12-year-old daughter—both with AgCC and intact AC—were able to read aloud the whole words and never read the 3-letter parts. Further, a 20-year-old college sophomore with AgCC in Sperry (1968) not only read midline-divided words holistically, but also wrote them without hesitation with either hand. In contrast, typical readers, according to split fovea theory, process each half of words separately before their integration (Ellis & Brysbaert, 2010; Strother et al., 2017). That is, visual information about the letters falling to the left of a fixated word is projected initially to the RH while visual information to the right of fixation is projected to the LH. The two word-parts are then reunited in the LH, by transfer of information through the corpus callosum, before their recognition—clearly an impossibility for individuals with AgCC.

### Reading Development in Hebrew

In contrast to learning the rightward orthography of English, it is not necessary to inhibit ipsilateral RH-to LH innervation in learning the leftward orthography of Hebrew. Accordingly, there is evidence suggestive of AC mirror-to-non mirror transfer of kinesthetic letter engrams in Israeli children learning to read their native language. For example, during tachistoscopic presentation of concrete three-letter Hebrew nouns, Silverberg et al. (1980) found a RH (left visual field) advantage among second graders (about age seven) and a LH (right visual field) superiority for the same words among third graders (about age eight). This reversal of hemispheric dominance was accompanied by virtually no change to RH presentations but a dramatic 150 ms reduction in relative response times to LH presentations, suggestive of whole word decoding. A comparable leap from spelling-to-sound translations in grade 1 children to whole word (lexico-morpho-orthographic) processing in grade 2 children was considered by Share & Bar-On (2018) to be a “watershed” (p.448) or “turning point” (p. 449) in children’s Hebrew reading development. Share and Shalev (2004) speculated that this abrupt gain in reading proficiency grew out of a critical volume of print experience. Alternatively, in the present view, it may represent AC-mediated, mirror-to-non mirror-letter engram connectivity to ATL whole word processing following the development of visual-proprioceptive integration between 7-8-years of age. In accord, visual-spatial working memory for Hebrew words predicts Hebrew reading comprehension in grade 2 and in grade 5 (Nevo & Bar-Kochva, 2015).

In contrast to English orthography, the right to left order of ipsilateral kinesthetic letter engrams is congruent with Hebrew orthography. This may explain why letter-sound learning in grade 1 facilitates orthographic learning of English (Ehri & Saltmarsh, 1995; Reitsma, 1983) but fails to do so in in the leftward orthography of Hebrew (Share & Shalev, 2004). That is, letter-sound inhibition of ipsilateral RH-mediated right to left ordering is productive only in rightward language orthographies. For example, Share (2004) found that Hebrew children with the poorest orthographic learning in grade 1 were also the best at “[S]low, laborious, letter-by-letter decoding” (p. 293) via the supplementary vowel pointing that, in Hebrew, makes an orthographic string transparent in terms of grapheme-phoneme correspondences. These grade 1 students were “clearly processing print in a very different manner” (p. 289) than late grade 2 students who discarded supplementary vowel points for a phonologically under-specified script that highlights lexical, morphological, and orthographic processing (Share & Bar-On, 2018). This laborious, letter-by-letter decoding in grade 1 may reflect mirror-opposition between vowel-pointed script and ipsilateral kinesthetic letter engrams that disappears in the second grade with the development of AC mirror-to-non mirror (RH-to-LH) letter engram access to ATL wh ole words.

The foregoing account of beginning reading development in Hebrew may apply to right eye/LH-dominant but not to left eye/RH-dominant children. Reading disability in Hebrew, as in English, is associated with LH-mediated phonological impairment, although manifested by difficulty with vowel-pointed script rather than grapheme-phoneme correspondences (Schiff et al., 2012). Selective impairments in either reading rate or accuracy (Shany & Breznitz, 2011; Shany & Share, 2010) resemble the impairments found in English readers with dyslexia (Lovett, 1984; 1987). In both languages, left eye/RH-dominant beginning writers may be unable to phonologically cope with the mirror-conflict between visual and kinesthetic letter engrams. Consequently, they become “invisibly” handicapped by unwittingly mapping mirror-reversed kinesthetic letter engram innervation onto the visual letter shapes they are learning. Hypothetically, if writing instruction were delayed to 7–8 years of age, left eye/RH-dominant children learning both leftward and rightward orthographies would show a leap in reading comprehension comparable to that of right eye/LH-dominant beginning readers of Hebrew.

Interestingly, adults with severe reading rate difficulties, when reading in both English and Hebrew, show exceptional peripheral letter recognition (English to the right and Hebrew to the left) together with poor central letter recognition (Geiger & Lettvin, 1987; Geiger et al., 1992). This letter recognition pattern led to a novel remedial treatment with English dyslexic adults that involved practicing peripheral reading through an overlay containing a word-sized window while focusing centrally on a small cross to the left of the window (Geiger & Lettvin, 1987). This regimen greatly improved reading comprehension but required continued practice. Geiger and Lettvin (1987) concluded that “although normal readers generally learn to read in the foveal field, dyslexic readers learn to read outside the foveal field” (p. 1242). That is, adult readers with dyslexia may decode words in the same manner as the 5-year-old children who peripherally processed reading stimuli in the study by Holmes et al. (1977), cited in the introduction of this paper. Correspondingly, children with both reading difficulties and unstable binocular coordination (e.g., see Jainta & Kapoula, 2011) make fewer non-word reading errors with larger (vs. smaller) print size (Cornelissen et al., 1991) or with the left eye occluded (Cornelissen et al., 1992). The latter intervention may be equivalent to Geiger and Lettvin’s (1987) focus on the overlay cross. Both focusing on the overlay cross and occluding the left eye may reduce visual-proprioceptive asynchrony by encouraging peripheral right eye/LH access to ATL whole word processing. In accord, Hogg (1968) reported that a normally intelligent 11-year-old-boy, affected by severe dyslexia, shifted his eye preference from left to right when viewing objects beyond a distance of 18 inches.

## Conclusion and Directions for Further Research

The essential argument in this review has been that beginning writers of English must cope with unwanted ipsilateral RH-to-LH corpus callosal innervation that is kinesthetically reversed in order and orientation from the rightward direction of print. In right eye/LH-dominants, ipsilateral mirror-innervation is inhibited through letter-sound decoding. However, in left eye/RH-dominants, ipsilateral mirror-innervation is uninhibited and induces sequential (e.g., was/saw) and spatial (e.g., b/d) letter writing confusion that precludes normal reading development. Age-appropriate sight word decoding and reading comprehension in AgCC, despite impaired phonological awareness (Temple and Ilsley, 1994), suggests that the anterior commissure (AC) facilitates mirror-to-non mirror interhemispheric letter engram transfer. Hypothetically, the AC would normalize reading development in left eye/RH-dominants if letter writing instruction was postponed until maturation of visual-proprioceptive integration at 7–8 years of age. Congruently, Laszlo and Bairstow (1985) argued that dyslexia may develop as a secondary consequence of writing problems that stem, in turn, from underdeveloped kinesthesia. Laszlo and Bairstow (1984) further noted that kinesthesia and writing readiness develop naturally in most children by 7 years of age.

Of note, delaying letter writing instruction until 8 years of age was recommended over 50 years ago by the Dutch neuropsychiatrist P. Mesker (1969/1980; see an English summary in van Eyck, 1980) based on Mesker’s extensive clinical experience with Dutch children affected by learning disorders. Mesker claimed that writing before this age could result in manual-motor and ocular-motor habits that were detrimental to the development of reading and spelling proficiency. Mesker’s claim is supported by subsequent evidence of impaired bimanual coordination (Gladstone et al., 1989; Rousselle & Wolff, 1991) and binocular coordination (Jainta & Kapoula, 2011; Kirkby et al., 2011) in children with dyslexia. However, as Mesker’s clinical impressions lacked empirical validation, his work has received little attention outside of Holland (e.g., van Grunsven et al., 2003, 2009).

On a final note, in a tractography study of 50 prematurely born sixteen-year-old adolescents, Northam et al. (2012) found that language impairment was only detectable in 19 who showed reductions in bilateral AC temporal lobe size while there was evidence of a compensatory AC size increase among those without language problems. Northam et al. concluded that this finding “calls for a systematic investigation of interhemispheric connections in other developmental populations where LI [language impairment] is a predominant feature”. . . (p. 3795). Such an investigation would help to determine whether the AC enlargement in individuals with AgCC (e.g., de Vin & Ten Tusscher, 2019; Fischer et al., 1992; van Meer et al., 2016) correlates with reading comprehension and whether an increase in AC temporal lobe connectivity/size accompanies the second-grade leap in Hebrew reading proficiency.
